# Multi-Omics Approach to Evaluate Effects of Dietary Sodium Butyrate on Antioxidant Capacity, Immune Function and Intestinal Microbiota Composition in Adult Ragdoll Cats

**DOI:** 10.3390/metabo15020120

**Published:** 2025-02-11

**Authors:** Anxuan Zhang, Deping Li, Tong Yu, Mingrui Zhang, Yingyue Cui, Haotian Wang, Tianyu Dong, Yi Wu

**Affiliations:** 1 State Key Laboratory of Animal Nutrition and Feeding, College of Animal Science and Technology, China Agricultural University, Beijing 100193, China; shubai929@cau.edu.cn (A.Z.); 2022304010128@cau.edu.cn (T.Y.); zhangmingrui@cau.edu.cn (M.Z.); sy20233040846@cau.edu.cn (Y.C.); wanghaotian@cau.edu.cn (H.W.); dongtianyu@cau.edu.cn (T.D.); 2 Hangzhou Netease Yanxuan Trading Co., Ltd., Hangzhou 310051, China; lideping@corp.netease.com

**Keywords:** sodium butyrate, adult cat, antioxidant capacity, gut microbiota, immune function

## Abstract

Objectives: Sodium butyrate (SB) is a typical postbiotic known to positively affect economic animals in recent years, but research on SB in pet cats is scarce. Consequently, this study sought to explore the influence of SB on anti-inflammatory and antioxidant capacity, immune function, and gut microbiota of adult cats through the assessment of biochemical parameters and comprehensive integrative omics analysis. Methods: A total of 30 adult cats were divided into three groups: a basal diet (NC), basal diet with 0.05% SB (SB5), and basal diet with 0.1% SB (SB10). The experiment lasted for 6 weeks. Results: The results indicated that the fecal level of calprotectin was lower in the SB10 group than in the SB5 and NC groups. The SB10 group reduced the serum levels of TNF-α, IL-1β and DAO compared with the NC group (*p* < 0.05). In addition, the SB10 diet increased the GSH-Px level and decreased MDA content compared with the NC diet (*p* < 0.05). Transcriptomic analysis showed that the gene expression of *VCAM1* exhibited a notable decrease in the SB10 group compared to the NC group (*p* < 0.05). The analysis of gut microbiota revealed that the richness of gut microbiota was higher in the SB10 than in the NC group (*p* < 0.05), and the abundance of Lachnospiraceae, *Lachnoclostridium*, *Blautia*, and *Roseburia* was greater in the SB10 than in the NC group (*p* < 0.05). Conclusions: Dietary SB could enhance the antioxidant and anti-inflammatory capacity, improve immune function, and positively regulate the gut microbiota composition in adult cats.

## 1. Introduction

With the continuous development of the economy and the renewal of people’s concepts, pets are increasingly regarded as family members. Therefore, the impact of pet food on health and nutrition has attracted significant attention, particularly the improvement of antioxidant capacity and intestinal health. Under normal conditions, the antioxidant system maintains cellular homeostasis by scavenging excess reactive oxygen species. However, when reactive oxygen species are generated in quantities beyond the body’s capacity to neutralize them, oxidative stress occurs, which can result in cellular injury and contribute to cardiovascular disease, cancer, chronic inflammation of asthma, Parkinson’s disease, etc [1]. Additionally, increased oxidative stress is associated with the promotion of tissue damage and autoimmune disease progression [2]. For instance, a study has identified oxidative stress as a mediator for chronic kidney disease in pets, with levels peaking in early chronic kidney disease [3,4]. A study of cats also found a high correlation between oxidative stress and hypertrophic cardiomyopathy [5]. Studies have found that some dietary substances have antioxidant properties, such as plant extracts, probiotics, and their derivatives [6,7]. Therefore, how to relieve the oxidative stress of pets and improve their body health through diet management has become a hot topic in pet nutrition research.

Postbiotics, consisting of non-viable microbes and/or their derivatives, have been extensively studied in human and livestock nutrition, and their application has expanded to human food, animal feed, and the pharmaceutical industry [8]. Postbiotics can influence gut microbiota balance by promoting the activity of beneficial microbes and suppressing the proliferation of pathogenic bacteria and exhibit a positive role in improving anti-inflammatory properties and enhancing the gut barrier function [9]. Complex microbial communities inhabit the gut of animals and play a key role in promoting animal health via different mechanisms, including enhancing nutrient absorption, producing a range of metabolites, and regulating gut function [10,11]. In healthy cats, the dominant phyla within the core intestinal microbiota are Bacteroidetes, Fusobacteria, Actinobacteria, Firmicutes, and Proteobacteria [12]. Irregularities in the gut microbiota are linked to diseases in cats. For example, the increase in *Escherichia coli* adhering to the ileal mucosa has been found to have a significant correlation with mortality rates in kittens [13]. Moreover, alterations in the gut microbiota are believed to be associated with the advancement of diabetes in cats [14]. Therefore, regulating the balance of gut microbiota is of great significance for improving the health and well-being of cats, and postbiotics exhibit tremendous potential in enhancing antioxidant ability and feline health by altering the gut microbial profile.

Sodium butyrate (SB) is a kind of typical postbiotic that acts on the digestive tract and directly or indirectly supports the development and repair of intestinal epithelial tissue [15]. Recent findings have revealed the positive influences of dietary SB on economic animals. Research has shown that SB can promote mitochondrial homeostasis and alleviate intestinal barrier damage induced by deoxynivalenol in pigs [16]. Furthermore, SB has been demonstrated to enhance the antioxidant capacity, lower intestinal pH, stimulate the growth of intestinal epithelial cells, and control the colonization of harmful bacteria in broilers [6,17]. Moreover, preweaning bull calves that ingested dietary SB promoted gastrointestinal development, which was indicated by reducing inflammation, regulating nutrient metabolism, and improving microbial community functions [18,19]. Although SB has shown great potential as a feed additive for improving gastrointestinal health in animals, limited research exists on the impact and mechanism of SB on intestinal health and antioxidant capacity in pet cats. Thus, our study aimed to explore the impacts of SB on gut microbiota, immune reaction, and antioxidant capacity in adult cats. It is important to highlight that this research aims to investigate the practicality of incorporating SB into cat food. The established concentration points are insufficient to detect the dose-dependent effects of SB, serving only as a point of reference.

## 2. Materials and Methods

### 2.1. Experimental Animals and Feeding Regimens

The experimental protocols concerning dietary treatments and animal handling were sanctioned by the Institutional Animal Care and Use Committee of China Agricultural University (AW11404202-2-2).

Cats with chronic medical conditions, cats unable to consume food orally, and female cats in pregnancy or lactation were excluded from the study. No cats showed signs of chronic gastrointestinal issues, immune-mediated diseases, or allergies. Before the trial began, all cats underwent medical examinations to evaluate their eligibility for participation in the upcoming experiment.

A total of 30 adult Ragdoll cats (15 females and 15 males, 1~2 years old, 3.6~4.5 kg body weight) participated in the experiment. The 30 cats were separated into three distinct treatment groups, with each group comprising 5 males and 5 females (*n* = 10). The formal trial lasted 42 days, during which the dietary treatments were as follows: (1) NC group: a basal diet; (2) SB5 group: a basal diet with 0.05% SB; (3) SB10 group: a basal diet with 0.1% SB. The basal diet fulfilled the nutritional specifications set by the NRC (2006) for adult Ragdoll cats. Table 1 shows the nutrient concentrations and ingredients of the three diets. The Ragdoll cats were fed at 08:30 and 16:30 every day and drank water freely. In the course of the experiment, the mental state, appetite, and defecation patterns of each experimental animal were systematically monitored, while ensuring an adequate supply of drinking water. The animals were not permitted to leave their designated environment outside of the experimental procedures. Throughout the duration of the experiment, the animal facility was maintained with proper ventilation and cleanliness, with daily cleaning protocols implemented. Additionally, disinfection procedures were conducted in accordance with established epidemic prevention guidelines.

### 2.2. Experimental Design and Sample Collection

On d 42, blood samples (2 mL) were collected from the small saphenous vein. Some samples underwent transcriptome analysis in the form of whole blood. The remaining samples were kept in centrifuge tubes at room temperature for 35 min to facilitate coagulation, followed by centrifugation at 3000 rpm for 10 min at 4 °C to isolate the serum. The serum samples and whole blood samples were retained at −80 °C. On d 42, fresh feces were collected from each cat and then preserved in sterile tubes and preserved at −80 °C. Blood and fecal samples were obtained by operators who had received specialized training. Throughout the procedure, efforts were made to maintain a calm and gentle environment for the cat, and appropriate measures were taken to soothe the animal following the completion of the sampling process.

### 2.3. The Parameters of Intestinal Barrier, Inflammation, and Antioxidant Ability

Biomarkers of intestinal barrier status comprised diamine oxidase (DAO), zonulin, intestinal fatty acid-binding protein (i-FABP), lipopolysaccharide (LPS), fecal calprotectin, and α-1 antitrypsin (α1-AT). Inflammatory markers comprised interleukin-6 (IL-6), IL-1β, and tumor necrosis factor-α (TNF-α). Antioxidant ability indicators included glutathione peroxidase (GSH-Px), catalase (CAT), total antioxidant capacity (T-AOC), total superoxide dismutase (T-SOD), and malonaldehyde (MDA). CAT, GSH-Px, and SOD were assessed using biochemical kits, and other markers were assessed using ELISA kits. All kits were from Shanghai Enzyme-linked Biotechnology Co., Ltd., (Shanghai, China) and used according to the instructions supplied by the manufacturer.

### 2.4. Microbiota Analysis

Fecal samples were processed to extract total bacterial DNA using the QIAamp Fast DNA Stool Mini Kit (Qiagen, Hilden, Germany). Universal primers 341F (5′-CCTAYGGGRBGCASCAG-3′) and 806R (5′-GGACTACNNGGGTATCTAAT-3′) were employed to amplify the V3-V4 region of 16S rRNA. The amplified products were sequenced using the Illumina MiSeq platform, producing paired-end reads of 300 base pairs (bp). Operational taxonomic units (OTUs) were clustered from the optimized sequences at a 97% similarity threshold using UPARSE software (version 11). A confidence threshold of 0.7 was applied.

Based on the community abundance data collected, the Kruskal–Wallis H test, along with the Games–Howell post hoc test, was employed to assess the differences in species among the three groups of microbial communities. The significance level of the differences in species abundance was evaluated to identify bacteria that exhibited significant differences among the groups. Subsequently, at the family and genus levels, bacteria with higher relative abundance were selected for further analysis.

### 2.5. Transcriptome Sequencing

Total RNA was isolated from the whole blood of NC (42 d), SB5 (42 d), and SB10 (42 d) groups using the MJZol total RNA extraction kit (Majorbio, Shanghai, China) according to the manufacturer’s instructions. Following enrichment and fragmentation, mRNA was reverse-transcribed into cDNA. The purified double-stranded cDNA was utilized for library construction via PCR amplification, and sequencing was performed using the NovaSeq Xplus sequencer (Illumina, San Diego, CA, USA). Transcript expression levels were estimated using the transcripts per million reads method, and gene abundances were quantified with RSEM. DEGs among the NC, SB5, and SB10 groups were identified using DESeq2 software (version 1.42.0), with the following criteria: *p* < 0.05 and |log2FC| ≥ 1. Functional annotation analysis, including Gene Ontology (GO) and functional enrichment analysis through Kyoto Encyclopedia of Genes and Genomes (KEGG), was also performed.

### 2.6. Statistical Analysis

IBM SPSS Statistics 26 (Chicago, IL, USA) was used for analysis, and GraphPad Prism (version 8.3.0, San Diego, CA, USA) was used to create graphs to visualize the data. The R tool was used to plot the microbial sequencing data. The Kruskal–Wallis H test was employed to ascertain the microbial abundance. The other data were subjected to one-way ANOVA with Tukey’s post hoc test. Statistical significance was defined as *p* < 0.05. The data were expressed as the mean ± standard error of the mean (SEM).

## 3. Results

### 3.1. Serum Inflammatory Parameters

**Figure 1:**
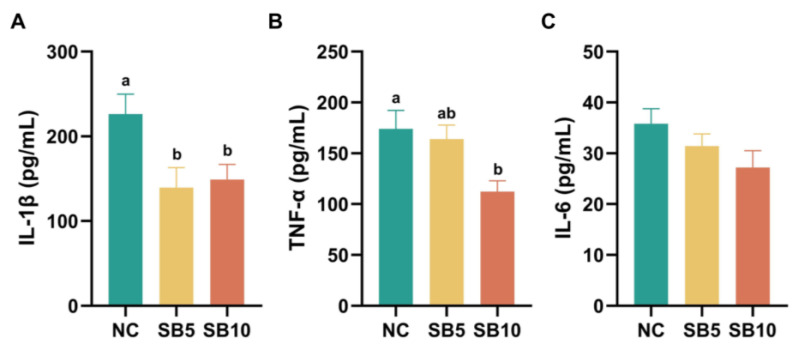
Serum inflammatory parameters in cats on d 42. (**A**): Interleukin-1β (IL-1β); (**B**): Tumor necrosis factor-α (TNF-α); (**C**): Interleukin-6 (IL-6). NC, a basal diet; SB5, 0.05% sodium butyrate supplemented on the basal diet; SB10, 0.1% sodium butyrate supplemented on the basal diet; *n* = 10. ^a,b^ Statistically significant differences are marked by distinct lowercase letters (*p* < 0.05). A *p*-value of less than 0.05 signifies a statistically significant difference.

Figure 1 displayed the inflammatory parameters in the serum of cats following different treatments. On d 42, the serum level of IL-1β was significantly lower in the SB5 and SB10 groups than in the NC group (*p* = 0.009 and 0.018; Figure 1A). Compared with cats consuming the NC diet, the TNF-α concentration in the serum significantly decreased in cats consuming the SB10 diet (*p* = 0.017), and no significant difference in the TNF-α concentration was observed between the SB5 and NC diets, nor between the SB10 and SB5 diets (*p* = 1.00 and 0.055; Figure 1B). There was no significant difference in serum IL-6 levels among the three dietary treatment groups (*p* = 0.13; Figure 1C).

### 3.2. Antioxidant Parameters

**Figure 2:**
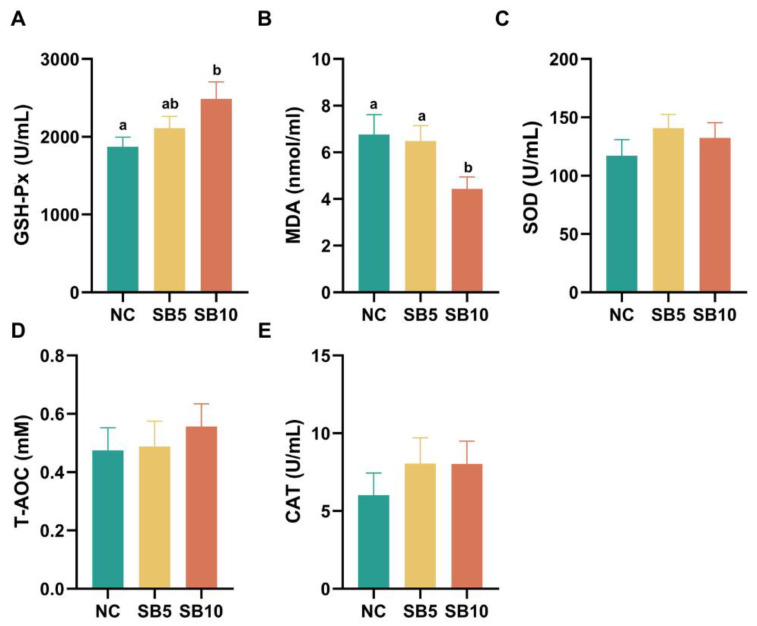
Antioxidant parameters in cats on d 42. (**A**): Glutathione peroxidase (GSH-Px); (**B**): Malondialdehyde (MDA); (**C**): Superoxide dismutase (SOD); (**D**): total antioxidant capacity (T-AOC); (**E**): Catalase (CAT). NC, a basal diet; SB5, 0.05% sodium butyrate supplemented on the basal diet; SB10, 0.1% sodium butyrate supplemented on the basal diet; *n* =10. ^a,b^ Statistically significant differences are marked by distinct lowercase letters (*p* < 0.05). A *p*-value of less than 0.05 signifies a statistically significant difference.

The antioxidant parameters of cats subjected to different treatments are presented in Figure 2. On d 42, compared with cats consuming the NC diet, the GSH-Px level increased in cats consuming the SB10 diet (*p* = 0.015), and there was no significant difference in the GSH-Px level in cats consuming the SB5 diet compared to cats consuming the NC and SB10 diets, respectively (*p* = 0.326 and 0.124; Figure 2A). The MDA level was lower in the SB10 group than in the NC and SB5 groups, respectively (*p* = 0.024 and 0.044; Figure 2B). No significant differences were observed in the serum levels of SOD, T-AOC, and CAT among the three dietary treatment groups (*p* = 0.43, 0.75, and 0.56; Figure 2C–E).

### 3.3. Intestinal Barrier Parameters

**Figure 3:**
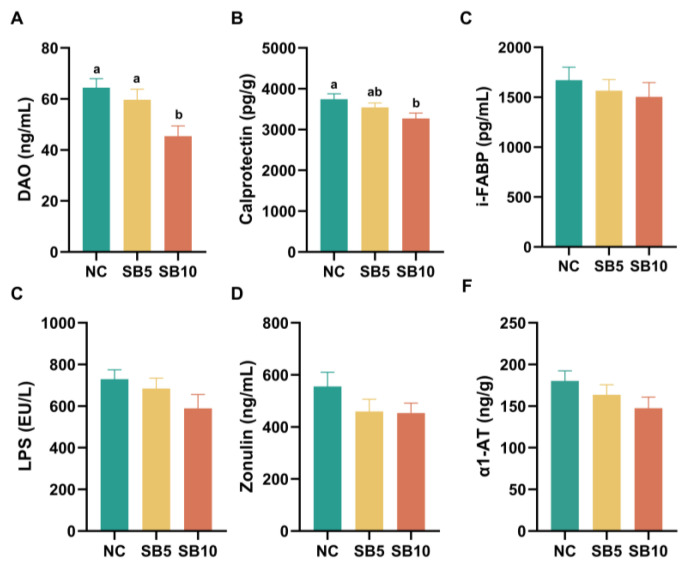
Intestinal barrier parameters in cats on d 42. (**A**): Diamine oxidase (DAO) in serum; (**B**): Calprotectin in feces; (**C**): Intestinal fatty acid binding protein (i-FABP) in serum; (**D**): Lipopolysaccharide (LPS) in serum; (**E**): Zonulin in serum; (**F**): α-1 antitrypsin (α1-AT) in serum. NC, a basal diet; SB5, 0.05% sodium butyrate supplemented on the basal diet; SB10, 0.1% sodium butyrate supplemented on the basal diet; *n* =10. ^a,b^ Statistically significant differences are marked by distinct lowercase letters (*p* < 0.05). A *p*-value of less than 0.05 signifies a statistically significant difference.

Figure 3 illustrates the parameters of the intestinal barrier in adult cats subjected to the different treatments. On d 42, the DAO level in serum was higher in the SB10 treatment, respectively, than in the NC and SB5 treatments (*p* = 0.002 and 0.015; Figure 3A). The fecal calprotectin content was lower in cats consuming the SB10 diet than in cats consuming the NC diet (*p* = 0.013) but was not different in cats consuming the SB5 diet compared to cats consuming the NC and SB10 diets, respectively (*p* = 0.271 and 0.140; Figure 3B). The levels of i-FABP, LPS, α1-AT, and zonulin in serum revealed no notable differences among the three dietary treatment groups (*p* = 0.65, 0.20, 0.21, and 0.24; Figure 3C–F).

### 3.4. Transcriptomic Analysis

**Figure 4:**
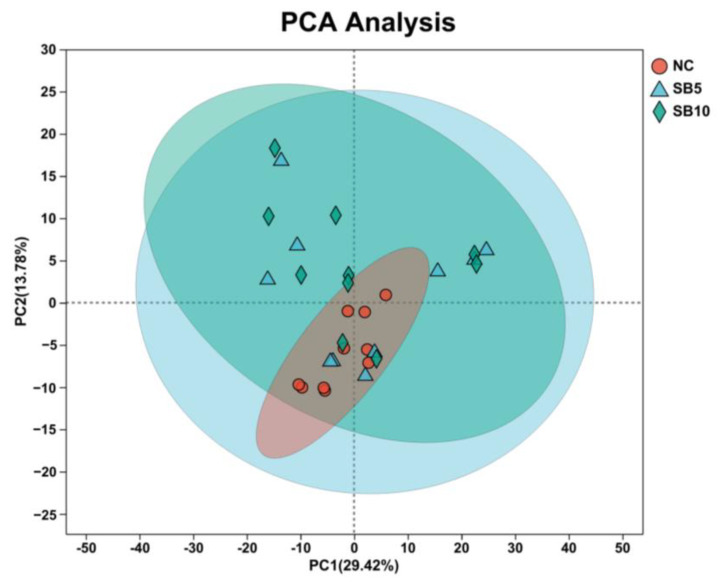
Separation of the gene expression patterns in the blood of cats among the NC, SB5, and SB10 groups.

A total of thirty libraries were established using mRNA extracted from the blood of cats in the NC, SB5, and SB10 groups, resulting in the acquisition of 44,982,396 to 64,925,184 reads through sequencing. The GC content was found to be between 47% and 52%, suggesting that the sequencing data met the quality standards required for further analyses. The PCA demonstrated a distinct separation between the NC and SB10 groups, indicating that the diet treatment of the SB10 group notably affected the gene expression patterns in the blood of cats (Figure 4).

**Figure 5:**
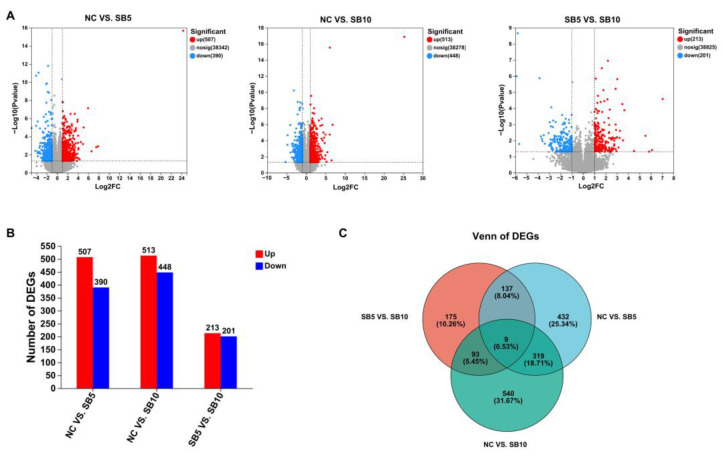
DEGs in the blood of adult cats from the different treatments. (**A**): Volcanic diagram of DEGs in the SB5 vs. NC comparison, the SB10 vs. NC comparison, and the SB10 vs. SB5 comparison. Each point corresponds to a distinct gene. The red point indicates a gene that is significantly up-regulated, the blue point denotes a gene that is significantly down-regulated, and the gray point signifies a gene with no significant difference. (**B**): Analysis of DEG quantity and expression in the transcriptome. (**C**): Venn plot illustrating the number of DEGs across three comparisons. NC, a basal diet; SB5, 0.05% sodium butyrate supplemented on the basal diet; SB10, 0.1% sodium butyrate supplemented on the basal diet; *n* = 10.

Through this analysis, we detected 507 up-regulated and 390 down-regulated DEGs in the NC group relative to the SB5 group. Additionally, there were 513 up-regulated DEGs and 448 down-regulated DEGs in the NC group relative to the SB10 group. Furthermore, the SB5 group exhibited 213 up-regulated and 201 down-regulated DEGs compared to the SB10 group (Figure 5A,B). The Venn diagram illustrated the overlap and distinct DEGs among the three groups (Figure 5C).

**Figure 6:**
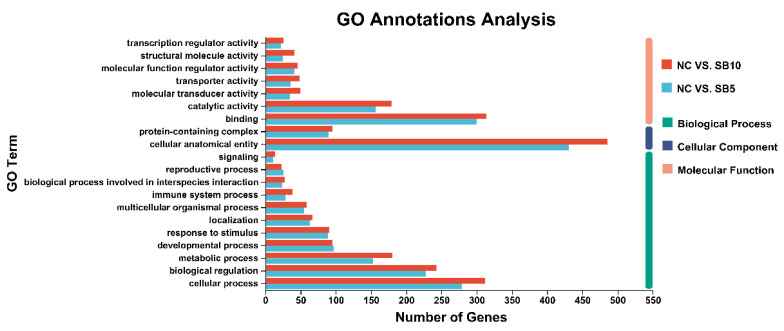
GO classification of DEGs in the NC vs. SB5 comparison and the NC vs. SB10 comparison. NC, a basal diet; SB5, 0.05% sodium butyrate supplemented on the basal diet; SB10, 0.1% sodium butyrate supplemented on the basal diet; *n* = 10.

The findings from the GO functional annotation analysis revealed the impacts of 0.05% and 0.1% SB supplementations on various subcategories of genes within the “biological process” (BP), “molecular function” (MF), and “cellular component” (CC) categories in cats (Figure 6). For the two comparison groups, in terms of BP, the GO term “cellular process” encompasses the largest number of genes; regarding CC, the GO term “cellular anatomical entity” contains the greatest number of genes; in the context of MF, the GO term “binding” has the highest count of genes.

**Figure 7:**
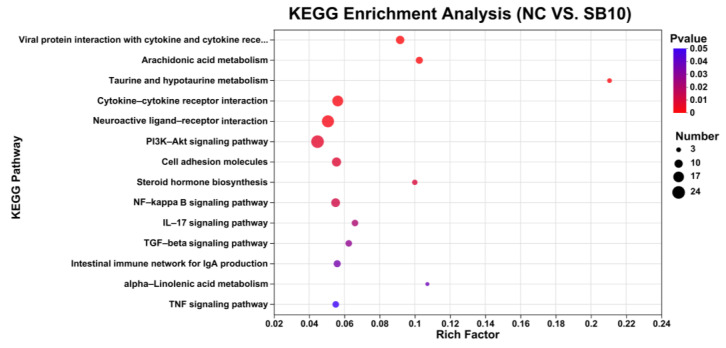
Enrichment analysis of DEGs in KEGG pathways in the NC vs. SB10 comparison. NC, a basal diet; SB5, 0.05% sodium butyrate supplemented on the basal diet; SB10, 0.1% sodium butyrate supplemented on the basal diet; *n* = 10.

KEGG enrichment analysis highlighted 36 signaling pathways enriched with DEGs in the comparison between the NC and SB10 groups (*p* < 0.05). Some pathways related to metabolism (arachidonic acid metabolism, taurine and hypotaurine metabolism, alpha-linolenic acid metabolism, steroid hormone biosynthesis), immunity (viral protein interaction with cytokine and cytokine receptors, TGF-beta signaling pathway, intestinal immune network for IgA production, neuroactive ligand–receptor interaction), inflammation (cytokine–cytokine receptor interaction, NF-kappa B signaling pathway, IL-17 signaling pathway, TNF signaling pathway, cell adhesion molecules), and oxidation (PI3K-Akt signaling pathway, TGF-beta signaling pathway, NF-kappa B signaling pathway) were selected (Figure 7). Most DEGs were enriched under the PI3K-Akt signaling pathway.

In comparison to the NC group, the alterations in part of DEGs within the SB10 group are also presented in Table 2. It was observed that certain DEGs participate in multiple pathways, suggesting their significant role in the regulation of physiological processes within the organism.

### 3.5. Effects of Sodium Butyrate on Fecal Microbial Community in Adult Cats

**Figure 8:**
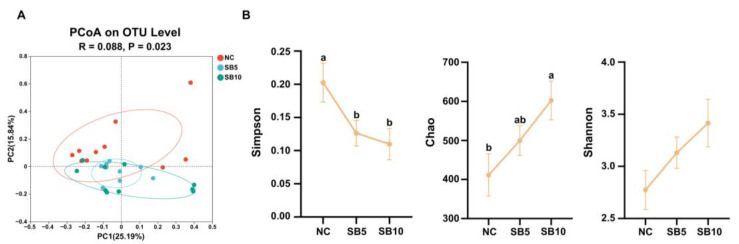
The β diversity and α diversity analyses of fecal microbiota among the three groups. (**A**): Principal component analysis at the OTU level. (**B**): The α diversity analysis employing the Simpson, Chao, and Shannon indices. NC, a basal diet; SB5, 0.05% sodium butyrate supplemented on the basal diet; SB10, 0.1% sodium butyrate supplemented on the basal diet; *n* = 10. ^a,b^ Statistically significant differences are marked by distinct lowercase letters (*p* < 0.05).

DNA sequences from the 30 fecal samples were analyzed, with an average length of 408 base pairs (bps). The PCoA revealed a clear separation in the clustering of fecal microbial communities at the OTU level among the three treatments (*p* < 0.05; Figure 8A). The Shannon index had no change among the three treatments (*p* ≥ 0.05; Figure 8B). A significant decrease in the Simpson index was observed in the SB5 and SB10 groups, respectively, compared to the NC group (*p* < 0.05; Figure 8B). In addition, the Chao index demonstrated a marked increase in the SB10 group compared to the NC and SB5 groups, respectively (*p* < 0.05; Figure 8B).

**Figure 9:**
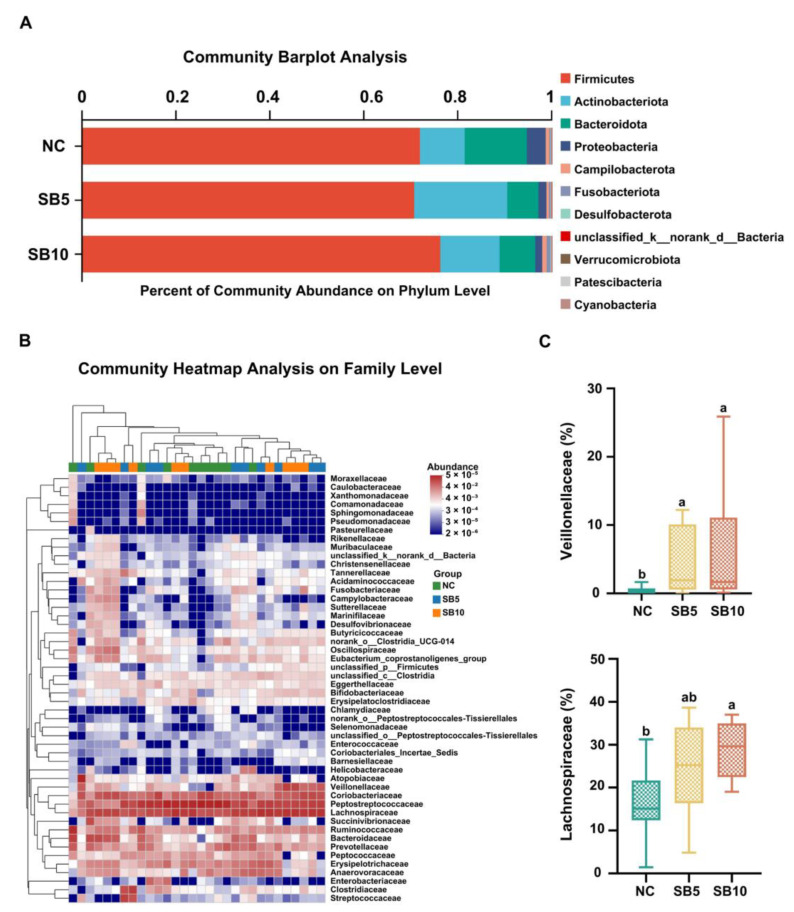
Variations in the fecal microbiota composition in adult cats subjected to different treatments on d 42. (**A**): The phylum-level abundance of fecal microbiota. (**B**): The family-level abundance of fecal microbiota. (**C**): The abundance of Lachnospiraceae and Veillonellaceae. NC, a basal diet; SB5, 0.05% sodium butyrate supplemented on the basal diet; SB10, 0.1% sodium butyrate supplemented on the basal diet; *n* = 10. ^a,b^ Statistically significant differences are marked by distinct lowercase letters (*p* < 0.05).

**Figure 10:**
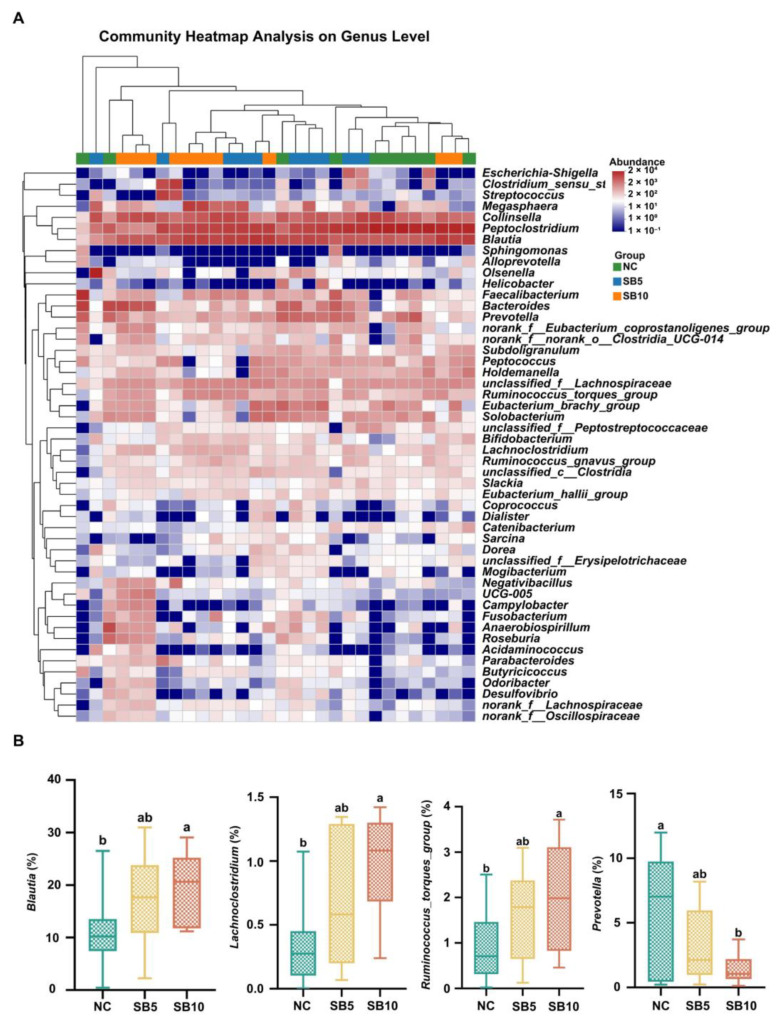
Changes in the composition of fecal microbiota in adult cats subjected to different treatments on d 42. (**A**): The genus-level abundance of fecal microbiota. (**B**): The abundance of *Blautia*, *Lachnoclostridium, and Roseburia*. NC, a basal diet; SB5, 0.05% sodium butyrate supplemented on the basal diet; SB10, 0.1% sodium butyrate supplemented on the basal diet; *n* = 10. ^a,b^ Statistically significant differences are marked by distinct lowercase letters (*p* < 0.05).

Figure 9 and Figure 10 illustrate the alterations in the fecal microbiota composition at the phylum, family, and genus levels in adult Ragdoll cats subjected to the different treatments. Figure 9A illustrates the bacterial composition at the phylum level on d 42. At the phylum level, the predominant bacterial groups identified were Firmicutes, Actinobacteria, Bacteroidetes, Proteobacteria, and Fusobacteria, while no statistically significant difference was observed in the abundance of these bacterial phyla among the different treatments. On d 42, at the family level, cats given the SB10 diet exhibited a greater abundance of Lachnospiraceae compared to cats given the NC diet. Additionally, the abundance of Veillonellaceae was increased in both the SB5 and SB10 groups compared with the NC group (*p* < 0.05; Figure 9C). At the genus level, relative to cats fed the NC diet, cats fed the SB10 diet showed a greater abundance of *Blautia*, *Roseburia*, and *Lachnoclostridium* (*p* < 0.05; Figure 10B).

### 3.6. Correlations Between Fecal Microbiota and DEGs

**Figure 11:**
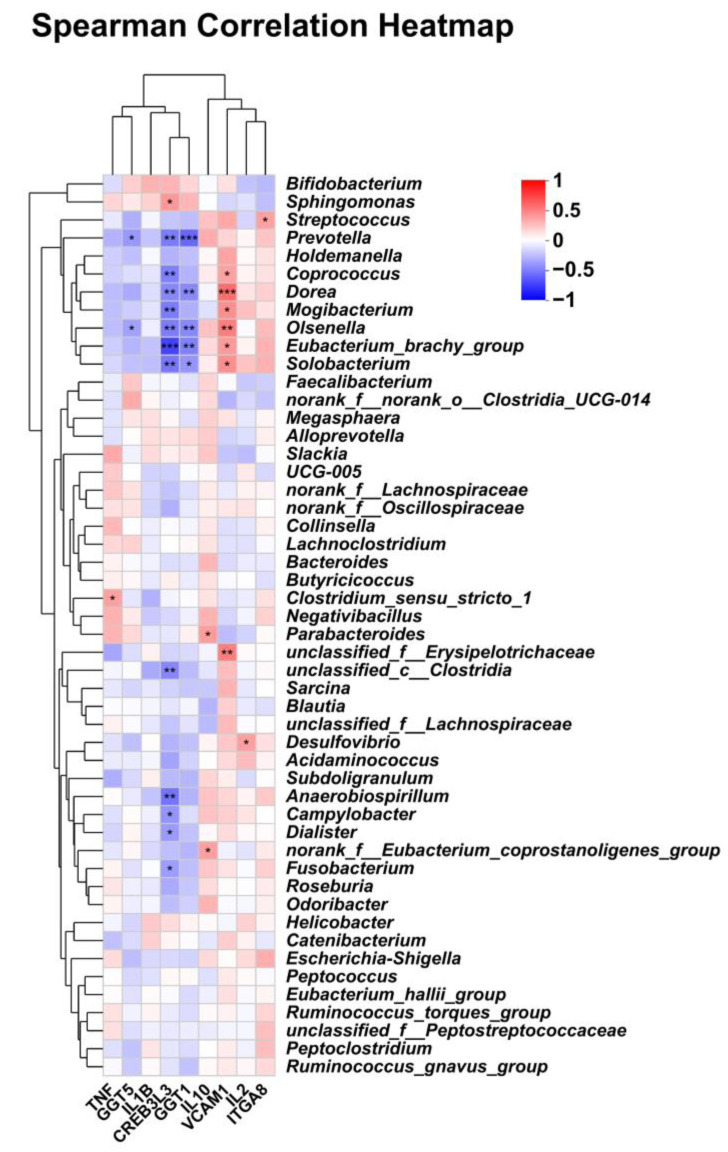
Correlation between selected DEGs and fecal microbiota at the genus level (*, *p* < 0.05; **, *p* < 0.01; ***, *p* < 0.001).

Spearman correlation analysis was conducted to detect the relationship between selected DEGs and microbiota at the genus level (Figure 11). The gene *VCAM1* was positively correlated with *unclassified_Erysipelotrichaceae*, *Solobacterium*, *Eubacterium_brachy_group*, *Oiseneila*, *Moqibacterium*, *Dorea,* and *Coprococcus* (*p* < 0.05). The gene *CREB3L3* was negatively correlated with *Fusobacterium*, *Dialister*, *Campyiobacter*, *Solobacterium*, *Eubacterium_brachy_group*, *Coprococcus,* and *Prevotella* (*p* < 0.05). There was a significant positive correlation between the gene *TNF* and *Clostridium_sensu_stricto_1* and between the gene *IL-10* and *norank_f_Eubacterium_coprostanoligenes_group* (*p* < 0.05).

## 4. Discussion

The excessive production of reactive oxygen species can induce oxidative stress and then result in disease and tissue damage [8]. How to relieve oxidative stress and improve body health through diet management has attracted significant attention in pet nutrition research. Complex microbial communities inhabit the gut of animals and support host health via various mechanisms, including optimizing nutrient absorption, producing a variety of metabolites, and regulating gut function [1,2]. Abnormal changes in the gut microbiota have been revealed to be linked to diseases in cats. Thus, regulating the balance of gut microbiota is important for improving the health and well-being of cats. Postbiotics exhibit a positive role in enhancing anti-inflammatory properties and improving the gut barrier function and can modulate the gut microbiota composition. Recent studies have revealed that SB, a kind of typical postbiotic, has positive effects on economic animal health and exhibits tremendous potential in enhancing antioxidant ability and feline health by altering the gut microbial profile. Therefore, our study aims to explore the effects of dietary SB on intestinal microbiota, immune reaction, and antioxidant capacity in adult Ragdoll cats.

Oxidative stress refers to a condition in which the generation of oxidants surpasses the organism’s capacity to counteract them with antioxidants, leading to a disruption in redox balance and subsequent damage to cells, tissues, and organs [20]. The extent of oxidative stress is influenced by the equilibrium between the efficacy of the antioxidant defense system and the production of ROS [21]. Research has indicated that, in cats, oxidative stress is linked to gastrointestinal diseases, decreased immune function, and behavioral abnormalities [22]. The body’s antioxidant defense system mainly involves antioxidant enzymes [23]. GSH-Px is recognized as a quintessential antioxidant enzyme that safeguards organisms against oxidative damage by catalyzing thiol cofactors to reduce detrimental hydroperoxides, and an enhancement in its activity contributes positively to the augmentation of antioxidant capacity [24,25]. Free radicals promote lipid peroxidation processes in organisms, and MDA is one of the end products of the peroxidation of polyunsaturated fatty acids in cells [26]. Research indicated that SB could improve the antioxidant capacity of older laying hens by decreasing the level of MDA and increasing the activity of GSH-Px in the liver and serum [27]. Furthermore, an investigation showed that SB intervention significantly activated the Nrf2 antioxidant pathway at the molecular level and improved the antioxidant capacity of obese rat tissues by mediating the up-regulation of GSH activity [28]. Similar to these results, in our study, 0.1% SB supplementation led to a marked increase in the serum GSH-Px level and a notable reduction in MDA content, which indicated that the dietary addition of SB had antioxidant properties through the modulation of oxidoreductase activity in Ragdoll cats.

Inflammation is tightly linked to the immunity process and oxidative stress [29,30]. The immune system safeguards the host from pathogens through the secretion of inflammatory cytokines, which facilitate the clearance of harmful agents. Nonetheless, an overproduction of pro-inflammatory cytokines may result in inflammation [31]. Butyrate has been demonstrated to possess anti-inflammation properties by regulating cytokine production and influencing the functionality of immune cells [32,33]. IL-1β and TNF-α are classic pro-inflammatory cytokines involved in the promotion of inflammation and the stimulation of immunocompetent cells [34]. A study has shown that SB can reduce intestinal inflammation induced by diabetes mellitus, and the concentrations of IL-1β and TNF-α in mice that ingested SB decreased [35]. Additionally, dietary SB supplementation significantly decreased the serum pro-inflammatory cytokines (IL-1β, IL-8, IL-6, and TNF-α) and suppressed inflammatory hypoxia-inducible factor 1α and its downstream response elements in the colon, ultimately decreasing the susceptibility to LPS-induced inflammatory response in piglets [36]. In this study, supplementing the diet with 0.1% SB resulted in a reduction in serum TNF-α and IL-1β levels, implying that SB could mitigate inflammation and positively influence the immune status of adult Ragdoll cats.

SCFAs, especially butyrate, play an important role in preserving the integrity of the intestinal barrier [37]. Butyrate has been demonstrated to improve intestinal barrier function via an IL-10 receptor-dependent mechanism that up-regulates the expression of mucin 2 [38,39]. Additionally, research has demonstrated that butyrate modulates the biological responses associated with intestinal health by binding to several specific G protein-coupled receptors and inhibiting histone deacetylases [40]. Calprotectin is primarily sourced from neutrophils, whose fecal level is proportional to the inflammation degree, and is commonly used to determine the degree of intestinal inflammation [41]. A study involving human participants has shown that the supplementation with SB can lead to a decrease in fecal calprotectin levels and has a positive effect on the circadian clock, inflammation, sleep, and life quality in patients with inflammatory bowel disease [42]. Additionally, research revealed that a dietary supplement with a natural mixture of quercetin, lentinula edodes, and bromelain increased antioxidant capacity and could improve gastrointestinal health in adult female dogs, which was accompanied by reductions in indole, N-methylhistamine, and fecal calprotectin [43]. Notably, calprotectin from the intestine may play a role in the pathogenesis of chronic enteropathies in felines; one study found that compared with healthy cats, cats with chronic inflammatory enteropathies had an increased fecal calprotectin concentration [44]. The level of DAO in blood is positively correlated with the permeability of the intestinal barrier and is used as an ideal indicator for evaluating intestinal barrier injury [45]. One study using a burn model in mice showed that the administration of SB could lower DAO content in plasma, inhibit the expression of inflammatory mediator high mobility group box-1 protein, and decrease oxidative stress and intestinal inflammatory responses, ultimately contributing to attenuating the severity of burn-induced intestine injury [46]. Another research revealed that the long-term supplementation of coated SB reduced intestinal damage in laying hens infected with *Salmonella enteritidis* and also decreased the level of DAO in serum [47]. Our findings revealed that cats in the SB10 treatment group exhibited significantly reduced fecal calprotectin and serum DAO levels relative to those in the NC group, demonstrating the favorable effects of a 0.01% SB dietary supplement on intestinal barrier health and immune performance.

The gut microbiota can exert beneficial influences on the health of the host by providing vitamins and nutrients, defending against intestinal pathogens, and regulating the immune system [48]. In this study, we found that the treatments with 0.05% SB and 0.1% SB in diet promoted an enrichment of the fecal microbiota and improved the composition of the microbial profile in adult Ragdoll cats. Lachnospiraceae, the dominant butyrate producer in the gut, can suppress colonic pathogens, produce anti-inflammatory cytokines, and maintain the antioxidant capacity of the host and has been considered to play a crucial role in promoting gastrointestinal status [49,50]. Research has found that a decreased diversity of the Lachnospiraceae family is associated with intestinal inflammation [51]. Another study demonstrated that mice with colitis had a lower abundance of Lachnospiraceae, while mice infused with SB had a higher proportion of Lachnospiraceae, which indicated that SB could improve intestinal dysbiosis and alleviate the inflammatory of colitis by increasing Lachnospiraceae abundance [52]. The genus *Lachnoclostridium* belongs to the family Lachnospiraceae [53]. One study found that fecal microbiota transplantation could alleviate histopathological changes and reduce the oxidative status and expression of key cytokine in the colon of mice with experimental coliti, which was accompanied by the up-regulation of *Lachnoclostridium* abundance [54]. *Roseburia* is one of the most abundant butyrate-producing bacteria in the intestine and has been shown to maintain energy homeostasis and prevent intestinal inflammation by producing beneficial metabolites [55]. Previous research has indicated that *Roseburia* possesses the ability to rectify the dysbiosis of an intestinal microbial community induced by fecal microbiota transplantation from donors with mastitis, which is possible by increasing the butyrate production and thus mitigating bacterial translocation and repairing the gut barrier [56]. *Blautia* is an anaerobic bacterium with probiotic properties and is widely found in the gut of mammals [57]. A study has shown that the addition of SB to the diet tends to promote the intestinal mucosal barrier function and enhance the growth performance of weaned piglets by altering the intestinal microbiota and increasing the abundance of *Blautia* [58]. In this study, in comparison to the NC group, the abundance of Lachnospiraceae, *Blautia*, *Roseburia,* and *Lachnoclostridium* was significantly increased in the SB10 group. These findings suggested that dietary SB could facilitate the proliferation of beneficial gut microbiota and help to foster better intestinal health in felines.

Notably, the 0.1% SB supplementation resulted in a rise in Veillonellaceae abundance in this study. Veillonellaceae is commonly found in the gut of humans, ruminants, and swine [59], but its function within the intestinal environment remains a subject of debate. In humans, findings found that Veillonellaceae was lower in the gut of depressed patients than in healthy people, and an increased proportion had a beneficial effect in the prevention of postpartum depression [60,61]. Nevertheless, a study conducted on human subjects indicated that the increase in Veillonellaceae was suggestively correlated with an increased risk of intrahepatic cholangiocarcinoma (ICC) [62]. A study on dogs indicated that the abundance of Veillonellaceae was greater in the intestinal flora of aged dogs compared to adult and juvenile counterparts [63]. Furthermore, research conducted on the rat colon has demonstrated that Veillonellaceae was capable of synthesizing significant quantities of short-chain fatty acids, including propionic acid and lactic acid. These short-chain fatty acids were recognized not only for their anti-inflammatory effects but also for their role as a primary energy source for colonic epithelial cells [64]. Some existing studies have indicated that an increase in the relative abundance of Veillonellaceae is advantageous for cats. A study revealed that lower levels of metabolites in detrimental processes were associated with an enhanced abundance of Veillonellaceae in the intestines of aged cats [65]. Research conducted on kittens found that the abundance of Veillonellaceae was significantly reduced within the intestines of those infected with fetal triceps, *Tritrichomonas foetus*, accompanied by colonocyte autophagy [66]. This variation in Veillonellaceae may be attributed to disparities among different species. On the whole, although Veillonellaceae is controversial in other species, in cats, studies have shown that an increase in the abundance of Veillonellaceae was advantageous for both kittens and old cats. In this study, based on the analysis of biochemical data, it can be seen that feeding 0.1% SB can improve immunity, antioxidant levels, and inflammation in adult cats. Previous research conducted on rats suggests that Veillonellaceae may bolster the anti-inflammatory properties in adult cats through the production of short-chain fatty acids; however, the precise mechanisms underlying this effect warrant further investigation.

To better understand the mechanisms driving these biochemical and physiological changes in adult Ragdoll cats, we conducted an analysis of the blood transcriptome and analyzed the correlation between the microbiota and transcriptomic data. The transcriptome analysis showed that multiple pathways, including the NF-kappa B signaling pathway, neuroactive ligand–receptor interaction, and PI3K-Akt signaling pathway, displayed a significant enrichment of DEGs after the 0.1% SB treatment. Among all DEGs, the genes *TNF*, *IL-1β,* and *VCAM1* were involved in various pathways, suggesting that these pathways had a series of effects on physiological processes within the organism. IL-1β and TNF-α exhibit significant pro-inflammatory properties and are capable of stimulating the release of numerous pro-inflammatory indicators [67]. In this study, relative to the NC treatment, the serum levels of TNF-α and IL-1β were decreased in the SB10 treatment, but KEGG analysis of the transcriptome indicated significant up-regulations of *TNF* and *IL-1β* in the SB10 group. Conflicting results between transcriptomes and proteomes have also been observed in the studies of other investigators [68,69,70]. Indeed, the dissociation of translation from transcription is a characteristic observed in IL-1β and TNF-α [71,72]. Specifically, with regard to IL-1β and TNF-α, although a significant amount of corresponding mRNAs are generated during transcription, these mRNAs are degraded when adequate translation signals are not provided [73]. This could result in inconsistent alterations in the mRNA and protein levels of TNF-α and IL-1β. In this study, it is suggested that TNF-α and IL-1β may be controlled by a separate mechanism after their transcription into mRNA. This could lead to a situation where not all of these mRNAs are translated into their respective proteins, resulting in a discrepancy between transcription and protein levels. The exact mechanisms of this process, including how translation signals are regulated and the conditions that trigger these signals, require further detailed investigation to clarify. Furthermore, the VCAM1 protein, classified within the immunoglobulin superfamily, is stimulated by inflammatory mediators, and its overexpression can facilitate the evasion of tumor cells from the immune system response and inhibit the activity of ROS [74,75]. Research has found that butyrate not only supported the overall catalytic activity of GPx to arrest vascular smooth muscle cell proliferation but also inhibited the expression of NF-kappa B target inflammatory genes, including inducible nitric oxide synthase, cyclooxygenase-2, and VCAM-1 [76]. Similarly, in this study, a dietary addition of 0.1% SB was found to improve antioxidant capacity by increasing the level of GSH-Px and concurrently reducing the expression of the *VCAM1* gene in adult cats, indicating that SB had the potential to mitigate oxidative stress in Ragdoll cats.

Gastrointestinal signs, such as vomiting, diarrhea, or anorexia, are one of the common reasons cat owners make unconventional appointments with vets [77]. This study focused on how SB supplementation impacts antioxidant levels, immune response, and gut microbiota in adult Ragdoll cats, with the goal of assessing its potential use in commercial cat food. The findings of our study indicate that incorporating 0.1% SB into the diet of adult Ragdoll cats can markedly enhance their immune function and intestinal health. This evidence suggests that the inclusion of SB as a dietary additive in commercial cat food may contribute to a decrease in the incidence of gastrointestinal disorders in felines from a nutritional perspective, thereby reducing the veterinary expenses for cat owners. It is worth noting that while this study has established the viability of SB as an additive in cat food, numerous issues remain to be addressed in its practical application. For instance, while a concentration of 0.1% SB has demonstrated beneficial effects in Ragdoll cats, the potential advantages of higher concentrations remain uncertain. The duration of this experiment was 6 weeks. In previous animal studies that utilized an SB diet, the duration of the experiments varied, with the shorter period being 1 week and the longer extending to 12 weeks [78,79]. Consequently, in the context of the current experiment, an extension of the experimental duration may yield more favorable outcomes. Over time, adult Ragdoll cats are likely to acclimate to a diet with SB, resulting in the stabilization of their biochemical indicators within a defined range. Furthermore, whether the reaction of different species of cats to SB is similar also needs to be further explored. For example, under identical experimental conditions, it remains unclear whether significantly altered gut bacteria in other feline species are consistent with those in Ragdoll cats. Additionally, the underlying mechanisms of the dissociation between mRNA expression and protein production of TNF-α and IL-1β require further investigation. It is worth noting that the response of pets to different diets based on sex is an important consideration. We additionally conducted a two-way ANOVA to evaluate the effects of sex, diet, and their interaction. However, no significant effects were observed for sex or the sex–diet interaction, which might be due to the small number of animals in each treatment group in the study. In future studies, we will focus on the gender-specific responses of pets to dietary changes.

## 5. Conclusions

In summary, the addition of SB in the diet increased anti-inflammatory and antioxidant capacities and improved the intestinal barrier function in adult Ragdoll cats. In addition, dietary SB could modulate the gut microbiota composition by boosting the abundance of Lachnospiraceae, *Blautia*, *Lachnoclostridium,* and *Roseburia*, and regulate the expression of genes related to inflammation and oxidative stress. These results lend scientific credibility to the use of SB in pet food to improve the intestinal health and welfare of companion animals.

## Figures and Tables

**Table 1:** The ingredient and nutrient content of the basal diet.

Items	Content
Ingredient, %	
Fish meal	6.00
Poultry fat	9.00
Fish oil	3.50
Rice	5.00
Salt	0.35
Potato	3.00
Potato starch	18.00
Sweet potato	4.50
Premix ^1^	1.50
Magnesium sulfate	0.25
Choline chloride	0.40
Taurine	0.15
Chicken liver	2.50
Chicken meal	45.85
Total	100.00
Nutrient composition, %	
Ether extract	23.11
Crude protein	37.17
Ash	7.75
Dry matter	91.01
Total energy, MJ/kg	23.27

Note: ^1^ The premix provided the following amounts per kilogram of feed: vitamin A (15,000 IU), vitamin B_1_ (30 mg), vitamin B_2_ (28 mg), vitamin B_3_ (110 mg), vitamin B_5_ (85 mg), vitamin B_6_ (12 mg), vitamin B_12_ (0.19 mg), vitamin D_3_ (15 IU), vitamin E (75,300 IU), Fe (FeSO_4_) 50 mg, Zn (ZnSO_4_) 38 mg, Mn (MnSO_4_) 18 mg, Cu (CuSO_4_) 3 mg I confirm, I (CaI_2_) 40 mg, Ca (CaI_2_) 20 mg, Co (CoSO_4_) 0.10 mg, Se (Na_2_SeO_3_) 260 mg, and Na (Na_2_SeO_3_) 0.05 mg.

**Table 2:** Pathways used for analysis and DEGs involved in these pathways.

Pathways	*p*-Value	DEGs
TNF signaling pathway	0.047	***VCAM1*** (down), ***CREB3L3*** (up), ***TNF*** (up), ***IL1B*** (up);
TGF-beta signaling pathway	0.026	*LTBP1*, ***TNF*** (up), *NOG*, *DCN*, ***NEO1*** (up)**;**
Cytokine–cytokine receptor interaction	0.0018	*GDF11*, ***ACKR4*** (down), ***CXCL12*** (up), *TNFSF9*, ***TNF*** (up), *CD70*, *TNFRSF8*, ***CXCL14*** (up), ***IL1B*** (up), ***IL10*** (down), ***IL2*** (up), *TNFRSF12A*;
IL-17 signaling pathway	0.020	***TNF*** (up), *MMP13*, *MUC5AC*, ***IL1B*** (up);
Intestinal immune network for IgA production	0.032	***CXCL12*** (up), ***IL10*** (down), ***IL2*** (up);
Steroid hormone biosynthesis	0.009	*CYP11A1*, *HSD17B1*, *CYP7A1*, *CYP2D6*, *AKR1D1*;
Cell adhesion molecules	0.008	***VCAM1*** (down), *JAM2*, *LRRC4B*, *NECTIN1*, *CLDN11*, *SIGLEC1*, ***NEO1*** (up), *CDH5*, ***ITGA8*** (down);
PI3K-Akt signaling pathway	0.007	*TNC*, *COL9A2*, *THBS2*, *TNR*, *LAMC3*, ***CREB3L3*** (up), *LAMA5*, *KITLG*, *ANGPT4*, *FGFR2*, *EGFR*, *COL1A2*, *VWF*, *MAGI2*, *ERBB3***, *IL2*** (up), ***ITGA8*** (down), *IGF1*;
Alpha-linolenic acid metabolism	0.035	*FADS2*, ***PLA2G2C*** (down), ***PLA2G2F*** (down)**;**
NF-kappa B signaling pathway	0.011	***VCAM1*** (down), ***CXCL12*** (up), ***TNF*** (up), *CARD10*, ***IL1B*** (up);
Taurine and hypotaurine metabolism	0.001	***GGT1*** (up), ***GGT5*** (up), *CDO1*;
Arachidonic acid metabolism	0.001	*LTC4S*, ***GGT1*** (up), ***PLA2G2C*** (down), *PTGES*, *PTGDS*, ***GGT5*** (up), ***PLA2G2F*** (down)**;**
Viral protein interaction with cytokine and cytokine receptor	0.0003	***ACKR4*** (down), ***CXCL12*** (up), ***TNF*** (up), ***CXCL14*** (up), *IL10*, ***IL2*** (up)**;**

DEGs in bold font are used for the correlation analysis of microorganisms and transcriptomes.

## Data Availability

Data are contained within the article.
